# Measuring sustainable employability: psychometric properties of the capability set for work questionnaire

**DOI:** 10.1186/s12889-022-13609-8

**Published:** 2022-06-14

**Authors:** Sait Gürbüz, Margot C. W. Joosen, Dorien T. A. M. Kooij, Arnold B. Bakker, Jac J. L. van der Klink, Evelien P. M. Brouwers

**Affiliations:** 1 Tranzo, Scientific Center for Care and Wellbeing, Tilburg School of Social and Behavioral Sciences, Tilburg University, Tilburg, The Netherlands; 2 International Business School, Hanze University of Applied Sciences, Zernikeplein 7, 9747 AS Groningen, The Netherlands; 3 Department of Human Resource Studies, Tilburg University, Tilburg, the Netherlands; 4 Center of Excellence for Positive Organizational Psychology, Erasmus University Rotterdam, Rotterdam, The Netherlands; 5 Department of Industrial Psychology and People Management, University of Johannesburg, Johannesburg, South Africa; 6 Optentia, North West University of South Africa, Vanderbijlpark, South Africa

**Keywords:** Capability set for work questionnaire, Sustainable employability, Validity, Work engagement, CSWQ

## Abstract

**Background:**

The capability set for work questionnaire (CSWQ) is being used to measure the new model of sustainable employability building on the capability approach. However, previous studies on the psychometric properties of the instrument are limited and cross-sectional. This two-way study aimed to (1) evaluate the convergent validity of the CSWQ with the theoretically related constructs person-job fit, strengths use, and opportunity to craft and (2) test the predictive and incremental validity of the questionnaire for the well-established work outcomes, including work ability, work engagement, job satisfaction, and task performance.

**Methods:**

A representative sample of 303 Dutch workers, chosen with probably random sampling, were surveyed using a one-month follow-up, cross-lagged design via the Longitudinal Internet Studies for the Social Sciences panel. The convergent validity was assessed by exploring the strength of associations between the capability set for work questionnaire and the theoretically related constructs using Pearson’s correlations. The predictive and incremental validity was evaluated by performing a series of linear hierarchical regression analyses.

**Results:**

We found evidence of the convergent validity of the capability set score by moderate correlations with person-job fit, strengths use, and opportunity to craft (*r* = 0.51–0.52). A series of multiple regression analyses showed that Time 1 capability set score and its constituents (i.e., importance, ability, and enablement) generally had predictive and incremental validity for work ability, work engagement, job satisfaction, and task performance measured at Time 2. However, the incremental power of the CSWQ over and above conceptually related constructs was modest.

**Conclusions:**

The findings support the convergent, predictive, and incremental validity of the capability set for work questionnaire with not previously investigated work constructs. This provided further evidence to support its utility for assessing a worker’s sustainable employability for future research and practical interventions.

## Background

According to a recent United Nations report [1], within 30 years, 1 in 6 individuals in the world will be older than the age of 65. This trend clearly shows that the aging of the labor force and declining young workers participation will remain a growing concern for many Western countries [2]. Since an older workforce is more likely to suffer from age-related health problems, it is essential for organizations to keep aging workers employable in a sustainable way to diminish job burnout, sickness absenteeism, and personnel turnover [3]. The topic of sustainable employability is also important from a worker’s standpoint. Because job loss due to decreased employability frequently leads to poverty and subsequent impairment of (mental) health [4].

Unfortunately, there is no consensus on the conceptualization of SE in the literature as the term is complex and the concept is hard to measure. For example, building on the Ability-Motivation-Opportunity framework, Le Blanc et al. [5] addressed the topic as the “extent to which a worker is able, willing, and has opportunities to work now and in the future” (p.3). Centering on work value and macro factors, Deng et al. [6] defined SE as “the ability of individuals, who pursue work with high intrinsic value and avoid digital exclusion, to remain in employment during their lifetimes” (p. 6). Recently, using proximal constructs, Fleuren et al. [7] defined SE as an “individual’s ability to function at work and in the labor market, or their ‘employability’, which is not negatively, and preferably positively, affected by that individual’s employment over time” (p. 15) and proposed nine indicators reflecting health, well-being, and employability components to measure SE over time. However, the most comprehensive and frequently cited conceptualization of SE, integrating the values and abilities of the worker and the opportunities provided by the environment is proposed by Van der Klink et al. [8]. This conceptualization is used in the current study.

Building on Amartya Sen’s capability approach [9], Van der Klink et al. [8] formulated SE as follows (1): “Sustainable employability means that, throughout their working lives, workers can realize tangible opportunities in the form of a set of capabilities. They also enjoy the necessary conditions that allow them to make a valuable contribution through their work, now and in the future, while safeguarding their health and welfare. This requires, on the one hand, a work context that facilitates them, and on the other hand the attitude and motivation to exploit these opportunities” (p.74). After decades of doing research on SE from a medical perspective, particularly focusing on complaints, the capability approach has common roots with the emerging subfield of positive organizational psychology [10], thus providing promising new insights to truly advance our knowledge on SE. Subsequently, to operationalize and measure a set of capabilities mentioned in the above conceptualization, a new instrument, the capability set for work questionnaire (CSWQ) was developed [11]. This instrument comprises seven capabilities which are “the use of knowledge and skills, development of knowledge and skills, involvement in important decisions, building and maintaining meaningful contacts at work, setting your own goals, having a good income, and contributing to something valuable” [11] (p. 38). The questionnaire measures to what extent those seven capability aspects (a) are considered valuable by the worker (importance), (b) are enabled in the work context (enablement), and (c) can be achieved (ability). Based on this operationalization, if an employee finds a capability aspect important (a), is enabled (b), and is achievable (c), a capability aspect is considered part of the capability set [11]. Limited previous research found that having a larger capability set was related to better work performance, work ability; and to lower absenteeism and depression [11, 12].

Although this new SE instrument has merits to assess the capability set of workers and is embraced by several organizations (e.g., the Netherlands Society of Occupational Medicine), it also met some criticisms [13]. In their critical reflection paper, Fleuren et al. [13], for example, argued that the new model of SE “is based on the insufficiently tested assumption that achieving value in work inherently leads to SE” (p.1). Moreover, the scholars who developed this instrument called for future research on the predictive validity of the questionnaire [11]. Thus, more empirical evidence is needed to validate the CSWQ by using different validity types (i.e., convergent, predictive, and incremental validity) and more robust research designs [14].

In the framework developed by Van der Klink et al. [7], the capability set for work refers to an individual worker’s abilities on the one hand, but also to workplace opportunities to achieve valuable work goals. We argue that, in a broader sense, the capability set for work, person-job fit [15], the use of character of strengths (i.e., individual abilities that allow a person to perform at their best) [16], and the opportunity to craft (i.e., a person’s perceived opportunity to proactively shape his or her job environment) [17] are related constructs that aim to enhance the fit between person and job, which, in turn, yields optimized functioning at work. Thus, investigating the convergent validity of the CSWQ with those constructs would be relevant. The first aim of the present study is, therefore, to evaluate the convergent validity of the CSWQ by relating it to theoretically related constructs. More specifically, we hypothesize that the capability set for work will be positively correlated with person-job fit, strengths use, and opportunity to craft [18].

The second purpose of the current study is to test the predictive validity of the questionnaire for well-established work outcomes, including work ability, work engagement, job satisfaction, and task performance. Third, we aim to test the incremental validity of the CSWQ by exploring whether it explains unique variance in work outcomes over and above conceptually related constructs (i.e., person-job fit, strengths use, and opportunity to craft).

## Methods

### Study population

A total of 303 Dutch workers were recruited for the present study. Data were collected using a two-wave design with a one-month time lag in September and November 2021 via the Longitudinal Internet Studies for the Social Sciences (LISS) panel governed by CentERdata (Tilburg University). This panel is made up of a representative sample of Dutch people who attend monthly online surveys. A true random sampling technique was used for selecting panel members from the population registry. Every year, members of the panel participate in a longitudinal survey that contains a wide range of topics such as work attitudes, health conditions, income, political views, values [19]. The LISS panel may be accessed here for further details: www.lissdata.nl.

Previous studies on the trajectory of work values have implied that the relative importance of work attitudes might fluctuate over time depending on contexts as a result of daily activities and environmental stimuli [20, 21]. Thus, in the present study, we have used a short time lag of one month between the two waves to investigate the predictive and incremental validity of the CSWQ for work outcomes.

At the first wave (Time 1), an online questionnaire was sent to randomly selected members of the LISS panel who work at different organizations (*N* = 597). The online questionnaire was completed by 401 respondents (response rate = 67.2%). After dropping incomplete questionnaires, 364 usable surveys were obtained. At the second wave (a month later), a follow-up questionnaire was sent to those respondents, and 315 out of 364 employees completed the questionnaires (response rate = 86.5%). After removing incomplete and unmatched surveys, the final sample consisted of 303 employees who completed both questionnaires.

We have checked the minimum sample requirement to test our hypotheses by using Faul et al’s [22] G*Power tool (version 3.1.9.7). The analysis indicated that a sample size of 173 is adequate to detect a medium effect size [23] for linear multiple regression (α = 0.05, power = 0.95, predictors = 10). As a result, the acquired sample size of 303 at the second wave is sufficient to test the research hypotheses.

Drop-out analyses between Times 1 and 2 showed that there were no significant differences on main variables (e.g., capability set for work) between those who completed both surveys and those who left out prior to completing Time 2 questionnaires. Table 1 shows the sample characteristics and descriptive statistics of the study variables. Out of 303 respondents, 52% was male (*N* = 159), the mean age was 46.51 years (SD = 12.29), the mean organizational tenure (in years) was 13.19 years (SD = 11.55), and the mean weekly working hours was 31.21 (SD = 9.93). Regarding the educational level of the participants; most participants held an intermediate vocational degree or above (76.9%, *N* = 286). The majority of the participants were married (62%, *N* = 188). Most participants worked for a profit organization (56.8%, *N* = 172) and had a fixed contract (89.4%, *N* = 271).

**Table 1:** Characteristics of the sample

Characteristics	N	%	Mean	SD^**a**^
Gender (*N* = 303)				
Female	144	47.5		
Male	159	52.5		
Age (in years) (*N* = 303)			46,51	12.29
Marital status (*N* = 303)				
Married	188	62		
Separated, divorced, or widowed	31	10.3		
Never married	84	27.7		
Organizational tenure (in years) (*N* = 303)			13.19	11.55
Education (*N* = 302^b^)				
Primary school	9	3		
Intermediate secondary education	40	13.2		
Higher secondary education	20	6.6		
Intermediate vocational	91	30		
Higher vocational education	93	30.7		
University	49	16.2		
Average working hours/week			31.214	9.93
Job type (*N* = 303)				
Profit	172	56.8		
Non-profit	131	43.2		
Contract type (*N* = 303)				
Temporary	32	10.6		
Fixed	271	89.4		

^a^ *SD* standard deviation

^b^ Due to missing answers, information was not available for all participants

### Measures

*Capability sets* were assessed at Time 1 via the CSWQ developed by Abma et al. [11] based on the model of sustainable employability [7]. The CSWQ captures whether seven work aspects (e.g., “using of knowledge and skills in your work”), are considered valuable by the worker (A = importance), are enabled in the work context (B = enablement), and can be achieved (C = ability). For each of these seven capabilities, the worker is questioned (A) “How important is < the aspect > for you?’ (B) “Does your work provide the opportunities to achieve < the aspect >” and (C) “To what extent do you actually achieve <the aspect>?” on a scale from 1 = “not at all” to 5 = “a very large extent”. The overall capability set score was calculated by taking the average of the seven work capabilities. An individual capability is considered part of the capability set of an individual worker when scores of A, B, and C are greater than 3 [11]. For example, if a worker values the aspect “having meaningful social contacts” to a large extent, and simultaneously is able and enabled to a large extent, the aspect is considered to be part of the worker’s capability set.

*Person Job-fit* was measured at Time 1 using a validated six-item scale [24] scored on a five-point Likert scale (ranging from 1 = strongly disagree to 5 = strongly agree). The scale contains demands-abilities fit and needs-supplies fit aspect of person job-fit. An example item is “There is a good fit between the demands of my job and my personal abilities”.

*Strengths use* was rated at Time 1 using the six-item scale developed by van Woerkom et al. [25]. An example item is “I use my strengths in my work” (0 = “almost never” to 6 = “almost always”).

*Opportunity to craft* was examined at Time 1 using five items [18]. An example item is “At work I have the opportunity to adjust the number of tasks I carry out” (1 = “never” to 5 = “very often”).

*Work ability* was examined at Time 2 using a short reliable and valid two-item version of the Work Ability Index (WAI) [26]. The two items are “How do you rate your own current work ability in relation to the physical demands of the job?” and “How do you rate this employee’s current work ability with respect to the mental demands of the work?”. Previous research has reported that this brief version of the WAI is reliable and valid [27]. Participants could respond to both items using on a five-point scale (1 = “very poor” to 5 = “very good”).

*Work engagement* was measured at Time 2 with the three-item ultra-short Utrecht Work Engagement Scale (UWES) [28]*.* An example item is “At my work, I feel bursting with energy*”.* Responses were given on a five-point scale from 1 (“never”) to 5 (“very often”).

*Job satisfaction* was rated at Time 2 using one single item [29]: “Taking everything into consideration, I am satisfied with my job”. Items were rated on a seven-point scale 1 (“strongly disagree”) to 7 (“strongly agree’). Previous meta-analysis has showed that one-single item can be used for measuring the overall job satisfaction [30].

*Task performance* was measured at Time 2 with three items by combining self-rated, coworkers and supervisory rating scores [31]. Item includes “how would >you, your direct supervisor, and your colleagues > evaluate your current overall work performance?”. Items were rated on a five-point scale 1 (“very poor”) to 5 (“excellent”).

### Analytical strategy

Data were analyzed using SPSS for Windows (release 26.0). We checked the normality of the data by calculating *Z* scores (skewness and kurtosis statistics divided by their standard errors) for composite variables [32]. All *Z* scores were less than 3.29 (*p* < .01), indicating that the data did not violate the normality assumption [33]. Cronbach’s alpha was used for assessing the internal consistency of scales used in the study. The convergent validity of the CSWQ was assessed by exploring the strength of associations between the capability set for work questionnaire and theoretically similar constructs using Pearson’s correlations. The following thresholds were used to interpret strength of correlation: r ≤ ±0.3 = weak; 0.3 < r ≤ 0.7 = moderate; 0.7 < r ≤ + 1 = strong [32]. Predictive and incremental validity of the CSWQ was evaluated with a cross-lagged design (i.e., predictors measured Time 1, outcomes measured at Time 2) by performing a series of multiple linear regression analyses. This design is more relevant than cross-sectional design for assessing the predictive validity [34, 35]. Moreover, consistent with previous research [11], age, gender, and average weekly working hours were included as control variables. All reported *p* values were two-tailed with an accepted significance level of 0.05.

## Results

### Descriptive statistics

Descriptive statistics (included in capability set, the means, standard deviations, corrected item-total correlations) and Cronbach’s alpha coefficients of the scales are presented in Table 2. As seen in Table 2, “use of knowledge and skills” (in 68%) and “building and maintaining meaningful contacts at work” (in 57%) appeared as most often included capability in the capability set. Cronbach’s alpha coefficients of all scales are above 0.7, and the item-total correlation ranged from 0.62 to 0.85, revealing a satisfactory internal consistency [36].

**Table 2:** Descriptive statistics and Cronbach’s alpha of the scales. [SD = standard deviation]

Variables	Included in capability set (in %) ^**a**^	Mean	SD^**b**^	Corrected item-total correlation	Cronbach’s α
Capability set score ^c^ (average of the seven work values)		3.53	0.54		
Use of knowledge and skills	68	3.94	0.67		
Development of knowledge and skills	40	3.48	0.72		
Involvement in important decisions	34	3.29	0.81		
Building and maintaining meaningful contacts	57	3.71	0.75		
Setting your own goals	37	3.39	0.78		
Having a good income	37	3.43	0.74		
Contributing to something valuable	43	3.47	0.82		
Person-job fit		3.66	0.76	0.73–0.82	0.92
Strengths use		4.24	1.01	0.79–0.85	0.94
Opportunity to craft		3.34	0.73	0.65–0.71	0.87
Work engagement (Time 2)		3.69	0.70	0.62–0.74	0.81
Work ability (Time 2)		4.26	0.65	0.69	0.82
Task performance (Time 2)		3.73	0.63	0.74–0.81	0.88
Job satisfaction (Time 2) ^c^		5.57	1.21		

^a^ An individual work value aspect is considered part of the capability set if the important (A), enablement (B), and ability (C) scores are greater than 3

^b^ *SD* standard deviation

^c^ Cronbach’s alpha calculations for the CSWQ and job satisfaction were not run since the former is not a scale and the latter is measured with an overall item

### Convergent validity of the CSWQ

The results for the convergent validity of the CSWQ are shown in Table 3. All Pearson’s correlation coefficients between the seven individual capability aspects and the capability set score with person-job fit, strengths use, and opportunity to craft were positive and significant (*p* < 0.01). In line with our predictions, results in Table 3 revealed that the larger the capability set scores, the higher the scores were for person-job fit (*r* = 0.509, *p* < 0.01), strengths use (*r* = 0.509, *p* < 0.01), and opportunity to craft (*r* = 0.552, *p* < 0.01). In addition, in general, moderate positive correlations were found, ranging from 0.254 to 0.579, between the seven individual capability aspects and person-job fit, strengths use, and opportunity to craft. The strongest positive correlation was found between work value “Use of knowledge and skills” and person-job fit (*r* = 0.579, *p* < 0.01), while the weakest but still significant correlation was observed between work value “Having a good income” and strengths use (*r* = 0.254, *p* < 0.01).

**Table 3:** Convergent validity of the CSWQ with person-job fit, strengths use, and opportunity to craft (*N* = 303)

Variables	1	2	3	4	5	6	7	8	9	10
1. Capability set score										
2. Use of knowledge and skills	.718									
3. Development of knowledge and skills	.755	.580								
4. Involvement in important decisions	.731	.403	.504							
5. Building and maintaining meaningful contacts	.683	.452	.370	.455						
6. Setting your own goals	.779	.445	.557	.548	.407					
7. Having a good income	.615	.366	.358	.317	.296	.434				
8. Contributing to something valuable	.709	.436	.447	.382	.435	.472	.321			
9. Person-job fit	.509^b^	.579^b^	.388^b^	.282 ^c^	.296 ^c^	.312 ^b^	.279 ^c^	.433 ^b^		
10. Strengths use	.509^b^	.576 ^b^	.335 ^b^	.316 ^b^	.351^b^	.361 ^b^	.254 ^c^	.376 ^b^	.556	
11. Opportunity to craft	.552^b^	.444^b^	.391 ^b^	.430 ^b^	.315 ^b^	.452 ^b^	.364 ^b^	.362 ^b^	.499	.492

^a^ All correlations are significant at < 0.01 level (two-tailed)

^b^ Moderate correlation (± 0.3 < r ≤ ±0.7) between the CSWQ and the other constructs

^c^ Weak correlation (r ≤ ±0.3) between the CSWQ and the other constructs

### Predictive validity of the CSWQ

The results for the predictive validity of the CSWQ are presented in Table 4. A series of multiple regression analyses revealed positive associations between the capability set score (Time 1) and work ability (*β* = 0.291, 95% CI .22–.48), work engagement (*β* = 0.385, 95% CI .36–.62), job satisfaction (*β* = 0.354, 95% CI .56–1.03), and task performance (*β* = 0.246, 95% CI .16–.41) measured at Time 2. Subsequently, we tested the predictive power of the constituents of capabilities, namely importance (Score A), enablement (Score B), and ability (Score C) dimensions measured at Time 1. As can be seen in Table 4, each of the three constituents of capabilities (Time 1) had predictive power for all outcome variables measured at Time 2 (see Model 2, 3, and 4 in Table 4). Finally, further multivariate analyses were conducted to determine which individual capability facet (Time 1) had the highest predictive power for outcomes. These analyses revealed that “use of knowledge and skills in the work” has substantial predictive potential for all work outcomes. However, we did not observe any significant association between two individual capability aspects (i.e., involvement in important decisions and setting your own goals) and outcome variables (Time 2).

**Table 4:** Predictive validity of the CSWQ (*N* = 303)

Predictors measured Time 1	Time 2 Work ability			Time 2 Work engagement			Time 2 Job satisfaction			Time 2 Task performance		
	***β*** ^***f***^	***SE***	***95% CI***	***β***	***SE***	***95% CI***	***β***	***SE***	***95% CI***	***β***	***SE***	***95% CI***
Capability set score^a^	.291	.06	.22–.48	.385	.07	.36–.62	.354	.12	.56–1.03	.246	.06	.16–.41
Importance^b^	.278	.07	.20–.45	.239	.07	.16–.43	.152	.12	.09–.58	.269	.06	.18–.43
Ability^c^	.262	.06	.16–.40	.390	.06	.32–.56	.365	.11	.51–.93	.219	.06	.11–.34
Enablement^d^	.253	.06	.15–.38	.407	.06	.34–.57	.427	.10	.64–1.04	.185	.06	.07–.29
Use of knowledge and skills^e^	.187	.07	.04–.32	.262	.07	.12–.40	.250	.04	.07–.23	.174	.02	.01–.10
Development of knowledge and skills^e^	−.060	.07	−.19–.08	.079	.07	−.05–.21	.044	.04	−.05–.10	−.034	.02	−.05–.03
Involvement in important decisions^e^	−.013	.06	−.12–.10	.152	.06	−.04–.27	.109	.04	−.01–.12	.055	.02	−.02–.06
Building and maintaining meaningful contacts^e^	.134	.06	.00–.23	.009	.06	−.11–.13	.040	.04	−.04–.09	.066	.02	−.04–.04
Setting your own goals^e^	.048	.06	−.08–.16	−.048	.06	−.17–.08	.105	.04	−.01–.12	.004	.02	−.02–.04
Having a good income^e^	.133	.06	.00–.23	−.040	.06	−.16–.08	.116	.03	−.01–.13	−.025	.02	−.04–.03
Contributing to something valuable^e^	.005	.05	−.10–.11	.081	.06	−.03–.19	.173	.03	.02–.15	.098	.02	−.01–.06

^a^ Model 1 adjusts for gender (1-male, 2-female, age (in years), weekly working hours, and the capability set score.

^b^ Model 2 adjusts for gender (1-male, 2-female, age (in years), weekly working hours, the importance constituent of capabilities.

^c^ Model 3 adjusts for gender (1-male, 2-female, age (in years), weekly working hours, the ability constituent of capabilities.

^d^ Model 4 adjusts for gender (1-male, 2-female, age (in years), weekly working hours, the enablement constituent of capabilities.

^e^ Model 5 adjusts for gender (1-male, 2-female), age (in years), weekly working hours, and all capability aspects.

^f^ *β* is the standardized beta coefficient, *SE* standard error, *95% CI* 95% confidence interval.

### Incremental validity of the CSWQ

We predicted the capability set score (Time 1) would explain unique variance in the work outcomes measured at Time 2 over and above conceptually related constructs (i.e., person-job fit, strengths use, and opportunity to craft) measured at Time 1. To test our prediction on incremental validity, we performed a series of three-step linear hierarchical regression analyses. We again included the controls (i.e., age, gender, and weekly working hours) in the first step to control for their possible extraneous effects. Then, at the second stage, we entered similar constructs individually. At the final stage, we added the capability set score to explore its additive power on the work outcomes. The results of these regression analyses are presented in Table 5. As can be seen, the capability set score explained incremental variance in work ability, work engagement, and task performance (*ΔR*^*2*^ = 0.043, 0.024, and 0.25, *p* < 0.01, respectively), beyond the variance accounted for by person-job fit. In a similar vein, the capability set score explained incremental variance in work ability, work engagement, job satisfaction, and task performance (*ΔR*^*2*^ = 0.052, 0.047, 0.040, and 0.024, *p* < 0.01, respectively), beyond the variance accounted for by opportunity to craft. Finally, we observed that the capability set score explained incremental variance in work ability, work engagement, and job satisfaction (*ΔR*^*2*^ = 0.039, 0.050, and 0.041, *p* < 0.01, respectively), beyond the variance accounted for the strengths use. However, the capability set score did not explain unique variance in job satisfaction over and above person-job fit (*ΔR*^*2*^ = .005, *p* = 094). Likewise, it did not explain unique variance in task performance over and above strengths use (*ΔR*^2^ = .007, *p* = 0.123).

**Table 5:** Incremental validity of the CSWQ (*N* = 303)

Predictors measured at Time 1	Time 2 Work ability			Time 2 Work engagement			Time 2 Job satisfaction			Time 2 Task performance		
	***β***^***a***^	***SE***	***ΔR***^***2***^	***β***	***SE***	***ΔR***^***2***^	***β***	***SE***	***ΔR***^***2***^	***β***	***SE***	***ΔR***^***2***^
Step 1 Controls	**–**	**–**	.017	**–**	**–**	.021	–	–	.038 ^b^	–	–	.016
Step 2 Person-job fit	.115^b^	.05	.049^b^	.455^b^	.05	.281^b^	.583 ^b^	.07	.378^b^	.139	.05	.048^b^
Step 3 Capability set score	.238^b^	.08	.043^b^	.175^b^	.07	.024^b^	.085	.11	.005	.182^b^	.07	.025^b^
Step 1 Controls	**–**	**–**	.017	**–**	**–**	.021	–	–	.038 ^b^	–	–	.016
Step 2 Opportunity to craft	.028	.06	.031^b^	.224^b^	.06	.131^b^	.206^b^	.11	.111^b^	.107	.06	.042^b^
Step 3 Capability set score	.276^b^	.08	.052^b^	.263^b^	.08	.047^b^	.242^b^	.14	.040^b^	.187 ^b^	.08	.024^b^
Step 1 Controls	**–**	**–**	.017	**–**	**–**	.021	–	–	.038 ^b^	–	–	.016
Step 2 Strengths use	.110	.04	.052 ^b^	.231^b^	.04	.132^b^	.222 ^b^	.07	.116^b^	.283^b^	.04	.109^b^
Step 3 Capability set score	.234^b^	.08	.039^b^	.266^b^	.08	.050^b^	.239^b^	.14	.041^b^	.099	.07	.007

^*a*^*β* is standardized beta coefficient taken from the last step. *SE* standard error

^b^ *P* < 0.01

c *P* < 0.05

## Discussion

The present two-wave study aimed to evaluate the convergent, predictive, and incremental validity of the CSWQ, a newly developed measure of sustainable employability based on Sen’s [9] capability approach. First, we examined the convergent validity by examining the strength of associations between the CSWQ and person-job fit, person-organization fit, strengths use, and opportunity to craft. Second, we tested whether the CSWQ has predictive and incremental validity for work ability, work engagement, job satisfaction, and task performance.

The results provide fair evidence to demonstrate that the CSWQ has satisfactory convergent validity. More specifically, we found that the capability set score was moderately correlated with person-job fit, person-organization fit, strengths use, and opportunity to craft, supporting our hypotheses. We also observed that the capability set score measured at Time 1 positively predicted the outcome variables, work ability, work engagement, job satisfaction, and task performance measured at Time 2. Moreover, we explored that the constituents of capabilities (i.e., importance, ability, and enablement) had also separately predictive power for all outcome variables. Among the seven individual capability aspects, the “use of knowledge and skills at work” facet had the highest convergent validity with strengths use. In a similar vein, this facet had the strongest predictive power for all work outcomes. However, our multivariate analyses depicted that the predictive power of three individual capability facets (i.e., involvement in important decisions, building and maintaining meaningful contacts, and setting your own goals) for the outcome variables was limited and not significant.

Finally, we found that the capability set score, in general, explained unique variance in work outcomes over and beyond conceptually related constructs (i.e., person-job fit, strengths use, and opportunity to craft). It is important to note that although the incremental power of the CSWQ for the work outcomes was significant, the explained incremental variance by the CSWQ in work ability, work engagement, and task performance was relatively weak. Moreover, we observed that the CSWQ did not explain unique variance in job satisfaction beyond person-job fit and in task performance over and above strengths use. These results suggest that modest evidence is obtained regarding the incremental validity.

These findings reveal that the use of CSWQ as overall capability set score or a constituent of capabilities (i.e., importance, enablement, and ability) rather than individual capability facets might be more relevant for predicting crucial work outcomes. In their development and validation study of the CSWQ, Abma et al. [11] reported that the capability set score was positively correlated with work performance and work ability. Subsequently, Van Gorp et al. [12] found that larger capability set was associated with better work ability for both workers with multiple sclerosis and workers from the general population. Our results are in line with the findings of previous studies.

Although the notion of SE gained increased attention over the last two decades, it has been measured using proximal constructs such as work ability, vitality, perceived employability until recently [37]. The CSWQ is unique among other instruments in that it measures employees’ SE as set of seven capabilities. With this research, we contribute to the literature and expand the limited previous work on the capability approach by answering Abma et al’s [11] call to further examine the validity of the instrument. Since the CSWQ is a new tool to measure sustainable employability, such research is important to provide further evidence for validation of the instrument. The satisfactory convergent, predictive, and incremental validity of the CSWQ with not previously investigated work constructs has provided further evidence to support its utility for assessing a worker’s capability set for future research and practical interventions.

Previous research reported that in employees with Multiple Sclerosis a larger capability set was associated with better work outcomes [12]. Since we have found that constituents of capabilities were also relevant for predicting crucial work outcomes, with its emphasis on being able and enabled, the CSWQ may especially provide a useful tool for disabled workers who often are confronted with an overemphasis on their disability and not on their strengths (abilities) in the work environment. In line with Abma et al’s study [11], we recommend that organizations and practitioners can use the CSWQ tool in two ways to measure a worker’s SE: capability scoring and discrepancy scoring. The former is particularly useful to examine how well workers achieve their values and which factors are boosting or inhibiting their SE. The latter, discrepancy scoring, can be used to identify obstacles in the realization of specific work values in terms of personal and contextual conversion factors [see for details [11]].

Moreover, there is little attention to environmental constraints in the work environment (enablers or disablers). Additionally, because of its innovative and positive view on sustainable employment, it may prove valuable to use this instrument to identify factors and areas in populations where job retention is a problem, such as health care professionals, especially during times of crisis  [38, 39].

### Strengths, limitations, and further research

This is the first research providing evidence on convergent validity of the CSWQ with person-job fit, person-organization fit, strengths use, and opportunity to craft. In a similar vein, our study is important as being the first endeavor reporting the predictive and incremental power of the CSWQ for work ability, work engagement, job satisfaction, and task performance by utilizing a cross-lagged design. Another strength of this research is including a representative Dutch sample via the LISS-panel. The panel surveyed respondents using a true probability sampling technique. Despite those strengths, the study also has some weaknesses, however. First, all constructs were measured through the use of self-reported data. Given that some work outcomes such as task performance cannot be objectively rated by self-reports, future studies may use other sources (e.g., immediate supervisor’s rating). Second, we used a two-way cross-lagged design to diminish common-method bias and obtain more valid results for the predictive and incremental validity [34, 35]. Future research using full cross-lagged panel designs with at least three waves may try to get a better grip on the causal ordering of the variables [35]. Third, all participants surveyed in the current study were from the Netherlands. Thus, it is still unknown whether the CSWQ is a valid and reliable tool for other countries and cultures.

Fourth, although our sample was representative in terms of several aspects (i.e., gender, age), workers with a fixed contract were overrepresented in our sample (89.4%). Therefore, a study that reexamines our results regarding SE with workers SE with a more balanced sample may advance our understanding of the topic. Fifth, in the present study we have considered that person-job fit, strengths use, and opportunity to craft would be the best conceptually related constructs for the CSWQ. However, some other constructs (e.g., “meaning” and “competence” dimensions of Spreitzer’s constructs of psychological empowerment  [40]) could also be viewed as conceptually related constructs for the CSWQ, which need to be investigated in future research.

Above all, future research should expand and advance our current knowledge on the topic by investigating the relationships between the contextual, organizational, and individual level of conversion factors [8] and SE. For instance, exploring whether implementing high-involvement Human Resource Management practices  [41] and creating a supportive leadership culture  [42] at the workplace can enhance a worker’s sustainable employability is the next course of action, which will be our upcoming research endeavor.

## Conclusion

The present cross-lagged study revealed that the CSWQ is a useful instrument with satisfactory psychometric properties. The findings support the convergent, predictive, and incremental validity of the CSWQ with not previously investigated work constructs although its incremental power is relatively modest. This provided further evidence to support the utility of the CSWQ for assessing a worker’s SE for future research and practical interventions.

## Data Availability

Data are available on reasonable request. The data set used is available from the corresponding author on reasonable request.
